# Biologic Prescription Patterns and Biomarker Determinants of First‐Line Dupilumab in Danish Adult Patients With Severe Asthma and Comorbid Atopic Dermatitis

**DOI:** 10.1111/cea.70117

**Published:** 2025-07-15

**Authors:** Marianne Baastrup Soendergaard, Kjell Erik Julius Håkansson, Susanne Hansen, Anne‐Sofie Bjerrum, Johannes Martin Schmid, Sofie Lock Johansson, Linda Makowska Rasmussen, Barbara Bonnesen, Roxana Vijdea, Anna von Bülow, Niels Steen Krogh, Ole Hilberg, Simon Francis Thomsen, Charlotte Suppli Ulrik, Celeste Porsbjerg

**Affiliations:** ^1^ Department of Respiratory Medicine Copenhagen University Hospital – Bispebjerg Copenhagen Denmark; ^2^ Department of Dermatology Copenhagen University Hospital ‐ Bispebjerg Copenhagen Denmark; ^3^ Department of Respiratory Medicine Copenhagen University Hospital – Hvidovre Hvidovre Denmark; ^4^ Centre for Clinical Research and Prevention, Frederiksberg Hospital Copenhagen Denmark; ^5^ Department of Respiratory Diseases and Allergy Aarhus University Hospital Aarhus Denmark; ^6^ Department of Respiratory Medicine Odense University Hospital Odense Denmark; ^7^ Allergy Clinic Copenhagen University Hospital – Gentofte Hellerup Denmark; ^8^ Department of Respiratory Diseases Copenhagen University Hospital – Gentofte Hellerup Denmark; ^9^ Department of Respiratory Medicine Aalborg University Hospital Aalborg Denmark; ^10^ Zitelab ApS Copenhagen Denmark; ^11^ Department of Respiratory Medicine Sygehus Lillebaelt – Vejle Sygehus Vejle Denmark

## Abstract

**Background:**

Asthma and atopic dermatitis are common type‐2 (T2)‐driven diseases that often coexist, although different T2 biomarkers indicate disease severity in each condition. Multiple biologics target T2 inflammation, but only dupilumab has been approved for both diseases. Little is known about patients with severe asthma and comorbid atopic dermatitis, particularly regarding how this comorbidity influences the choice of biologic for severe asthma.

**Methods:**

We utilised the nationwide Danish Severe Asthma Register to characterise patients with severe asthma and comorbid atopic dermatitis, and describe prescription patterns of biologics in patients with and without comorbid atopic dermatitis. We compared biomarker determinants of first‐line dupilumab prescription between the two groups using a multivariate logistic regression model adjusting for age and sex.

**Results:**

We identified 1137 patients initiating a biologic for severe asthma, of whom 192 (17%) had comorbid atopic dermatitis. Patients with comorbid atopic dermatitis more often had childhood‐onset asthma with an allergic phenotype. The prescription patterns of biologics differed according to comorbid atopic dermatitis status, and patients with comorbid atopic dermatitis were more likely to switch (OR 3.02, *p* < 0.001). In patients with comorbid atopic dermatitis, elevated IgE was the strongest biomarker determinant of first‐line dupilumab prescription (OR 8.77, *p* < 0.001), whereas in patients without, it was elevated FeNO (OR 1.76, *p* = 0.03).

**Conclusion:**

Biologic prescription patterns in severe asthma vary according to comorbid atopic dermatitis status. In patients with comorbid atopic dermatitis, elevated IgE predicts first‐line dupilumab prescription, indicating that the severity of atopic dermatitis influences treatment decisions. Additional research is needed to explore the management of coexisting T2 diseases with biological therapies.

## Graphical abstract

Almost one in five patients receiving biological treatment for severe asthma has comorbid atopic dermatitis. Patients with comorbid atopic dermatitis represent a distinct phenotype with early‐onset, allergic asthma. Comorbid atopic dermatitis impacts the choice of biologic for severe asthma in real‐life practice. IgE, immunoglobulin E; FeNO, fractional exhaled nitric oxide.

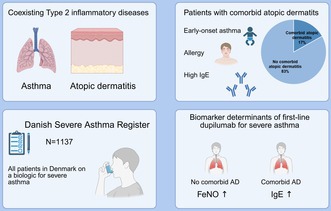

**Summary**

- Almost one in five patients receiving biological treatment for severe asthma has comorbid atopic dermatitis.
- Patients with comorbid atopic dermatitis represent a distinct phenotype with early‐onset, allergic asthma.
- Comorbid atopic dermatitis impacts the choice of biologic for severe asthma in real‐life practice.

## 1 Introduction

Asthma and atopic dermatitis are highly prevalent chronic inflammatory diseases that often coexist in the same patients [1]. Both diseases are multifactorial disorders influenced by genetic predisposition, environmental triggers, and dysfunction in skin and respiratory barrier integrity. Local anti‐inflammatory agents, such as inhaled corticosteroids (ICS) for asthma and topical corticosteroids or calcineurin inhibitors for atopic dermatitis, are the cornerstone of treatment for both diseases. Type‐2 (T2) inflammation plays a key role in both asthma [2] and atopic dermatitis [3], and key T2 cytokines such as interleukins (IL) 4, 5, and 13, and epithelial alarmins such as thymic stromal lymphopoietin (TSLP) and IL33 drive inflammation. Owing to the shared T2 inflammatory pathways, there are also shared T2 biomarkers between the two diseases. However, while blood eosinophil count and fractional exhaled nitric oxide (FeNO) are strong predictors of severity and exacerbation risk in asthma [4], total IgE count is the stronger predictor of severity in atopic dermatitis [5, 6].

As T2 inflammation is dominant in asthma and atopic dermatitis, biologics that target T2 inflammatory pathways are highly effective in both diseases. However, targeting the same T2 pathway is not always effective in both asthma and atopic dermatitis. For example, the anti‐IL13 biologic tralokinumab is effective in atopic dermatitis [7] and is approved for this indication, but tralokinumab failed as a treatment for asthma during phase II [8]. Conversely, the anti‐TSLP tezepelumab has proven effective in the treatment of severe asthma [9] but has shown limited efficacy in atopic dermatitis [10]. Notably, only one biological agent has received approval for use in both diseases: the anti‐IL4/IL13 dupilumab is effective at reducing exacerbation rates [11] and maintenance oral corticosteroid (mOCS) use [12] in severe asthma, and it also significantly reduces disease burden in atopic dermatitis [13].

Although asthma and atopic dermatitis frequently coexist and share an indication for dupilumab, there are no established guidelines for the simultaneous treatment of both conditions. This results in ambiguity in the choice of biologics for both dermatologists and respiratory physicians. Currently, it is unclear if and when dupilumab is the best choice for severe asthma with comorbid atopic dermatitis and vice versa. There is evidence to suggest an effect on asthma‐related outcomes when treating atopic dermatitis with dupilumab [14, 15, 16, 17], it is unknown whether there is a collateral effect on comorbid atopic dermatitis when treating severe asthma with dupilumab. It also remains unclear to what extent clinicians take comorbid atopic dermatitis into account when prescribing biological therapies for severe asthma in real life, and whether the presence of asthma similarly influences the choice of treatment for atopic dermatitis.

In this study, we aimed to examine the prevalence and clinical features of patients with comorbid atopic dermatitis in a nationwide cohort of patients receiving biologics for severe asthma. Furthermore, we aimed to investigate the real‐life prescription patterns of biological therapies in patients with severe asthma and comorbid atopic dermatitis. Finally, we aimed to identify biomarker determinants of first‐line dupilumab prescription, stratified by atopic dermatitis status.

## 2 Methods

We utilised data from the Danish Severe Asthma Register [18] (DSAR), a nationwide, complete cohort of patients with severe asthma treated with a biologic in Denmark. All patients commenced on a biologic are entered into the register, and data are registered at baseline, after 4 months of treatment, after 12 months, and then annually. Information on lung function, biomarkers, patient‐reported outcomes (PROMs), asthma medications, and demographics is entered into the register. DSAR is approved by the Capital Regions Centre for Knowledge (VD‐2018‐31), and all patients provided written informed consent.

### 2.1 Patient Population

In this study, we included all biologic‐naive patients who were started on a biologic for severe asthma between January 1, 2016, and January 31, 2024. In Denmark, the criteria for initiating a biologic are recurrent exacerbations (≥ 2 in the previous 12 months) or the need for mOCS more than 50% of the time despite high‐dose inhaled corticosteroid (ICS) therapy according to the ERS/ATS definitions [19] in combination with a second controller. Furthermore, patients should exhibit signs of relevant T2 inflammation for the individual biologics: sensitisation to a perennial allergen for omalizumab, blood eosinophil counts ≥ 0.3 cells x 10^9^/L or sputum eosinophils ≥ 3% for mepolizumab, benralizumab, and reslizumab, and FeNO ≥ 25 ppb or blood eosinophil counts ≥ 0.3 cells x 10^9^/L or sputum eosinophils ≥ 3% for dupilumab and tezepelumab. The commencement of a biologic for severe asthma is up to the treating asthma specialist, and there is free choice between all approved biologics.

In Denmark, dupilumab became available for the treatment of severe asthma on January 1, 2020, and therefore, the prescription patterns of biologics for patients with comorbid atopic dermatitis have been described before and after this date. We excluded patients initiated before January 1, 2016, as only omalizumab was available before this date.

The diagnosis of comorbid atopic dermatitis was obtained from DSAR. In DSAR, the diagnosis is physician‐reported at the time of initiation of therapy, and disease severity is not registered.

### 2.2 Statistical Analyses

The patients were categorised according to their atopic dermatitis status in DSAR. The baseline characteristics were compared using descriptive statistics. Continuous variables were compared using Student's T test or Mann–Whitney U test for parametric and non‐parametric data, respectively. Categorical data were compared using the chi‐square test or Fisher's exact test when appropriate. The treatment efficacy was assessed after 12 months using the paired t‐test or the Wilcoxon signed rank test for parametric and non‐parametric data, respectively. The McNemar's test was used to assess changes in categorical variables. Biomarker profiles were assessed as predictors of first‐line dupilumab prescriptions in multivariate logistic regression models adjusted for age and sex.

All *p*‐values were two‐sided and considered significant at *p* < 0.05. All data analyses and statistical procedures were performed using R statistical software (R version 4.3.0).

## 3 Results

**FIGURE 1:**
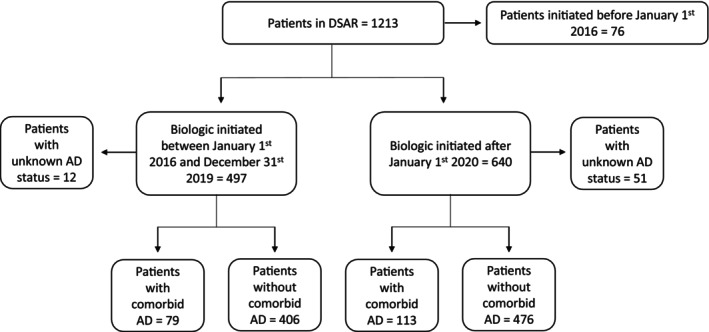
Flow chart.

In DSAR, we identified 1137 patients who commenced treatment with a biologic for severe asthma for the first time between January 1, 2016, and January 31, 2024 (Figure 1), of which 192 (17%) had comorbid atopic dermatitis. Of the 1137 included patients, 640 (56%) commenced treatment after January 1, 2020, when dupilumab became available for the treatment of severe asthma in Denmark.

### 3.1 Baseline Characteristics Differ According to Comorbid Atopic Dermatitis Status

**FIGURE 2:**
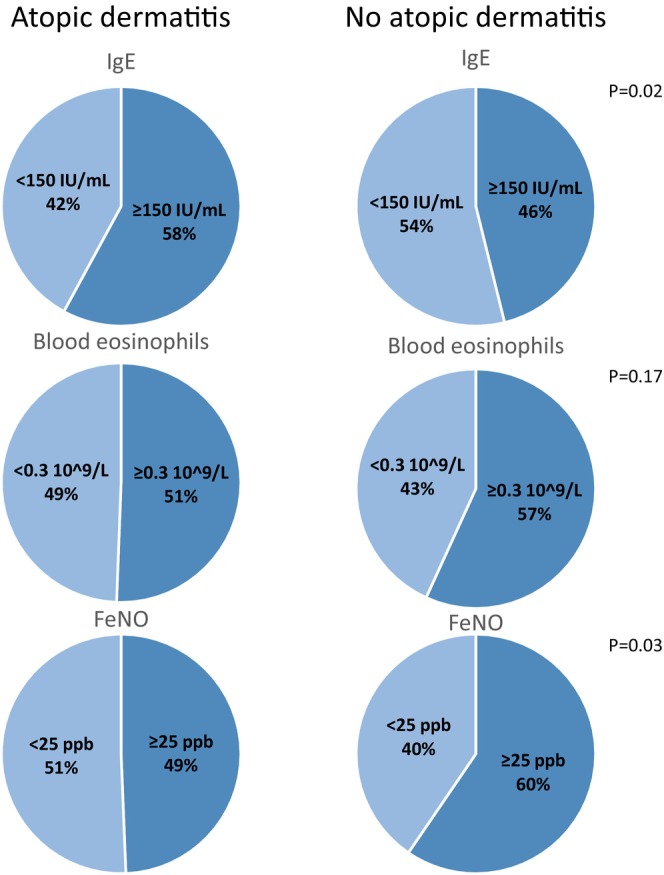
Baseline biomarkers in patients with and without comorbid atopic dermatitis.

Patients with severe asthma and comorbid atopic dermatitis differed significantly from those without comorbid atopic dermatitis across several clinical parameters (Table 1). Patients with comorbid atopic dermatitis were younger (49 vs. 56 years, *p* < 0.001) yet had asthma for a longer duration than those without comorbid atopic dermatitis (28 vs. 21 years, *p* < 0.001). Subsequently, a larger proportion of patients with comorbid atopic dermatitis had asthma debut during childhood than patients without comorbid atopic dermatitis (58% vs. 28%, *p* < 0.001). Fewer patients with comorbid atopic dermatitis had a history of smoking (38% vs. 48%, *p* = 0.02). A larger proportion of patients with comorbid atopic dermatitis had allergic sensitisation (76% vs. 48%, *p* < 0.0001) and allergic rhinitis (81% vs. 47%, *p* < 0.001). A larger proportion also had chronic rhinosinusitis (69% vs. 58%, *p* = 0.004); however, similar proportions had nasal polyps. Patients with and without comorbid atopic dermatitis were similar in terms of lung function, ACQ, and registered mOCS use, and exacerbations prior to the commencement of biological therapy. The differences in biomarker profiles between patients with severe asthma and comorbid atopic dermatitis are summarised in Figure 2. More patients with comorbid atopic dermatitis had elevated total IgE counts than patients without comorbid atopic dermatitis (58% vs. 46%, *p* = 0.02), whereas fewer had elevated FeNO levels (49% vs. 60%, *p* = 0.03).

**TABLE 1:** Baseline characteristics.

	Atopic dermatitis, *N* = 192	No atopic dermatitis, *N* = 882	*p*
Age, years	49 (15)	56 (14)	< 0.0001
Female sex, *n* (%)	113 (59%)	453 (51%)	0.07
BMI, kg/m^2^	28 (6)	27.7 (5.6)	0.50
Duration of disease, years	28 (21)	21 (18)	< 0.0001
Duration of disease > 10 years, *n* (%)	100 (72%)	404 (64%)	0.08
Age at onset, years	21 (20)	35 (20)	< 0.0001
Onset during childhood ≤ 18 years, *n* (%)	94 (58%)	201 (28%)	< 0.0001
Late onset ≥ 40 years, *n* (%)	31 (22%)	290 (45%)	< 0.0001
No of exacerbations in the year before biologic	3.23 (3)	3.17 (2.9)	0.80
Budesonide equvilant dose, mcg	1600 (1600–2000)	1600 (1496–2296)	0.82
Registered mOCS use, *n* (%)	41 (21%)	239 (27%)	0.13
Median dose	10 (5–12.5)	10 (5–12.5)	0.37
Biologic			
Mepolizumab, *n* (%)	75 (39%)	506 (57%)	< 0.0001
Reslizumab, *n* (%)	3 (2%)	15 (2%)	
Benralizumab, *n* (%)	17 (8%)	98 (11%)	
Omalizumab, *n* (%)	36 (19%)	81 (9%)	
Tezepelumab, *n* (%)	3 (2%)	20 (2%)	
Dupilumab, *n* (%)	58 (30%)	162 (19%)	
Later switch, *n* (%)	71 (37%)	184 (21%)	< 0.0001
FEV1, L	2.31 (0.89)	2.25 (0.87)	0.46
FEV1, % of predicted	70 (21)	71 (21)	0.85
FEV1/FVC	0.68 (0.14)	0.65 (0.15)	0.03
ACQ	2.7 (1.29)	2.49 (1.17)	0.09
Blood eosinophils, cells × 10^9^/L	0.3 (0.13–0.56)	0.34 (0.16–0.65)	0.17
IgE, IU/mL	224 (61–623)	128 (46–344)	0.007
IgE ≥ 150 IU/mL, *n* (%)	76 (58%)	287 (47%)	0.02
FeNO, ppb	24 (11–43)	32 (16–59)	0.002
Smoking status			
Never, *n* (%)	119 (62%)	454 (52%)	0.02
Former, *n* (%)	67 (35%)	400 (46%)	< 0.001
Current, *n* (%)	5 (3%)	14 (2%)	
Packyears	13 (11)	18 (17)	
Allergic sensitisation	104 (76%)	276 (48%)	< 0.0001
Allergic rhinitis, *n* (%)	155 (81%)	435 (47%)	< 0.0001
Chronic rhinosinuitis, *n* (%)	132 (69%)	509 (58%)	0.004
Nasal polyps, *n* (%)	82 (43%)	385 (44%)	0.85
Aspirin sensitivity, *n* (%)	16 (8%)	71 (8%)	1
Bronchiectasis, *n* (%)	46 (25%)	213 (25%)	1
Vocal cord dysfunction, *n* (%)	6 (3%)	15 (2%)	0.31
ABPA, *n* (%)	14 (7%)	28 (3%)	0.01
EGPA, *n* (%)	5 (3%)	31 (4%)	0.67
Dysfunctional breathing, *n* (%)	22 (12%)	71 (8%)	0.15
COPD, *n* (%)	29 (15%)	183 (21%)	0.09
GERD, *n* (%)	70 (37%)	278 (32%)	0.19
Cardiovascular disease, *n* (%)	55 (26%)	280 (32%)	0.17
Diabetes, *n* (%)	26 (14%)	80 (9%)	0.08
OSAS, *n* (%)	31 (16%)	92 (10%)	0.03

*Note:* Values are mean (SD) or median (p25–p75), unless otherwise stated.

### 3.2 Prescription Patterns of Biologics Are Different in Patients With Comorbid Atopic Dermatitis

**FIGURE 3:**
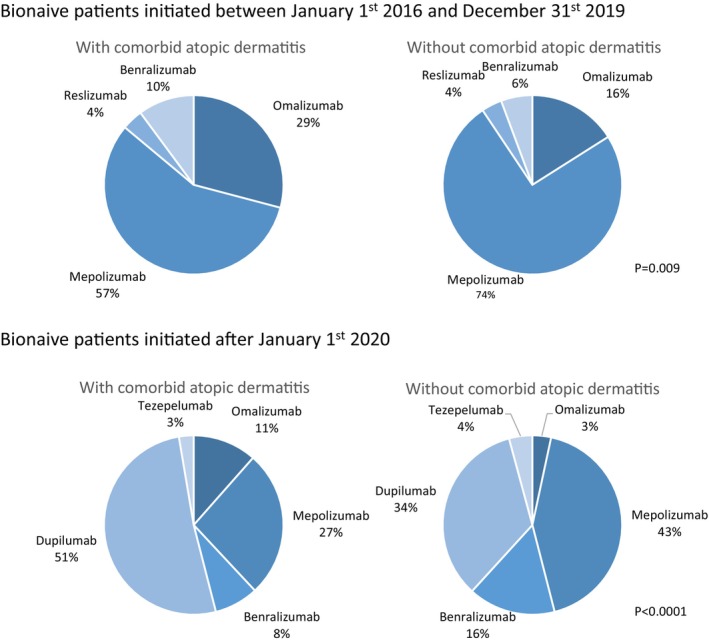
Prescription patterns of biologics in patients with and without comorbid atopic dermatitis.

The differences in the prescription patterns of biologics before and after the introduction of dupilumab are summarised in Figure 3. Before dupilumab became available for the treatment of severe asthma, patients with comorbid atopic dermatitis were more often prescribed first‐line omalizumab treatment than those without comorbid atopic dermatitis (29% vs. 16%, *p* = 0.01). Although more frequently used as a first‐line treatment in patients without comorbid atopic dermatitis, mepolizumab was the most frequently prescribed first‐line biologic in both groups before dupilumab became available. This changed after January 1, 2020, when dupilumab became the most commonly prescribed first‐line biologic therapy for patients with comorbid atopic dermatitis. It was the first‐line biologic for 51% of patients with comorbid atopic dermatitis and only for 34% of patients without atopic dermatitis. In patients without atopic dermatitis, anti‐IL5 biologics were the first‐line treatment in 59% of cases, whereas they were only prescribed as the first‐line treatment in 35% of patients with comorbid atopic dermatitis. Even after the introduction of dupilumab, patients with comorbid atopic dermatitis were more often prescribed omalizumab as first‐line treatment than patients without comorbid atopic dermatitis (11% vs. 3%, *p* < 0.0001).

**FIGURE 4:**
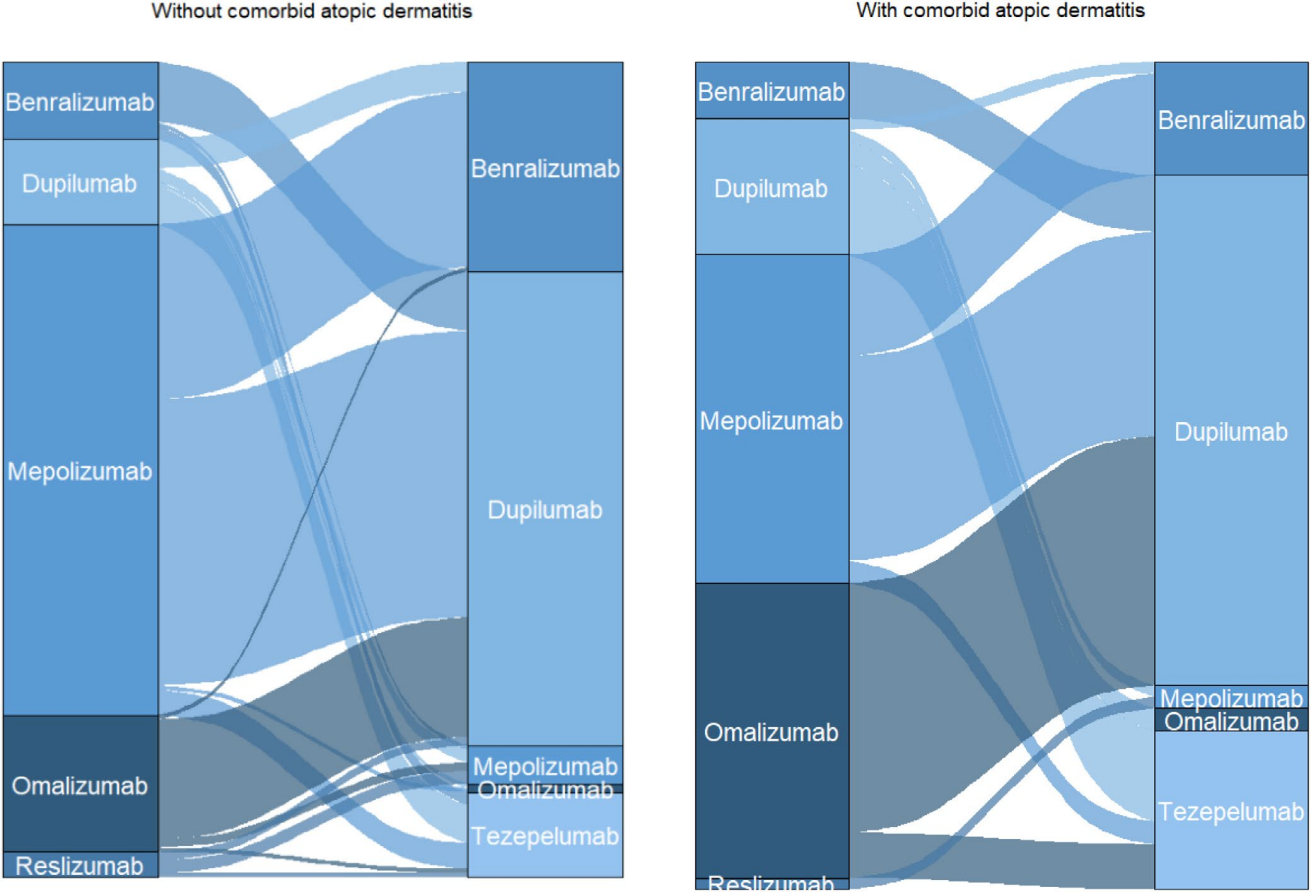
Switch patterns from first to second drug in switchers with and without comorbid atopic dermatitis during the entire follow‐up period.

Patients with comorbid atopic dermatitis were more likely to switch biologic during the entire follow‐up period compared to patients without atopic dermatitis, with 71 (37%) patients with comorbid atopic dermatitis switching biologic vs. 184 (21%) of patients without comorbid atopic dermatitis. In a logistic regression model adjusted for age and sex, the odds ratio for switching was 3.02 (1.62–5.73, *p* < 0.001) in patients with comorbid atopic dermatitis compared to those without. Switch patterns occurring after dupilumab became available are summarised in Figure 4. Dupilumab was the most frequent second drug, regardless of atopic dermatitis status.

### 3.3 Different Biomarkers Predict First‐Line Dupilumab According to Comorbid Atopic Dermatitis Status

We assessed how biomarker profiles determined first‐line dupilumab prescription in patients with and without comorbid atopic dermatitis initiated on a biologic after January 1, 2020, when dupilumab became available for severe asthma (Table 2). We found that in patients without comorbid atopic dermatitis, elevated FeNO levels were the determinant of the first‐line prescription of dupilumab; however, this was not the case in patients with comorbid atopic dermatitis. Elevated FeNO levels did not predict first‐line dupilumab prescription; however, patients with elevated total IgE were more likely to receive first‐line dupilumab compared to patients without elevated total IgE (OR 8.77, CI 2.96–29.7, *p* < 0.001).

**TABLE 2:** Biomarker predictors^a^ of first‐line dupilumab prescription for severe asthma in patients with and without comorbid atopic dermatitis initiated after January 1, 2020.

	Atopic dermatitis			No atopic dermatitis		
	OR	95% CI	*p*	OR	95% CI	*p*
FeNO ≥ 25 ppb	1.06	0.31–3.5	0.92	1.76	1.06–2.99	0.03
IgE ≥ 150 IU/mL	8.77	2.96–29.7	< 0.001	1.22	0.76–1.98	0.41
Blood eosinophils ≥ 0.3 e^9^/L, *n* (%)	0.54	0.15–1.78	0.32	0.99	0.61–1.63	0.99

^a^ Multivariate logistic regression models adjusted for age and sex.

### 3.4 Efficacy of Dupilumab in Asthma Outcomes Is Comparable to Other Biologics in Patients With Comorbid Atopic Dermatitis

We compared the efficacy of dupilumab with that of other biologics on asthma outcomes after 12 months of treatment in the subgroup of patients with comorbid atopic dermatitis (Table 3). We found that all patients showed similar improvements in asthma‐related clinical outcomes. Unsurprisingly, we observed differences between dupilumab and other biologics in terms of their effects on the biomarkers. Patients treated with biologics other than dupilumab experienced a larger decrease in blood eosinophil count (*p* < 0.001), and we also observed that patients on dupilumab experienced significant reductions in total IgE levels compared with patients on other biologics (*p* < 0.001).

**TABLE 3:** Outcomes for patients with severe asthma and comorbid atopic dermatitis initiated after January 1, 2020, after one year of treatment with biologics.

	Dupilumab, *N* = 48	Other biologics, *N* = 126	P‐value delta between groups
*Clinical outcomes*			
No of exacerbations in the previous 12 months			
At baseline	3.63 (2.67)	2.69 (2.5)	0.18
At 12 months	0.70 (1.13)	0.56 (1.07)	
*p*	< 0.0001	< 0.0001	
Registered mOCS use			
At baseline	10 (21%)	30 (24%)	0.08
At 12 months	8 (17%)	17 (13%)	
*p*	0.61	0.004	
ICS			
At baseline	1600 (1600–2400)	1600 (800–1600)	0.83
At 12 months	1600 (1600–1600)	1600 (800–1600)	
*p*	0.31	0.45	
FEV1, L			0.19
At baseline	2.15 (1.03)	2.26 (0.8)	
At 12 months	2.22 (1)	2.5 (0.86)	
∆ from baseline	0.06	0.20	
*p*	0.48	< 0.0001	
FEV1, % of predicted			
At baseline	65 (23)	69 (19)	0.15
At 12 months	69 (23)	78 (20)	
∆ from baseline	3 (14)	7 (15)	
*p*	0.24	< 0.001	
FEV1/FVC			
At baseline	0.66 (0.15)	0.67 (0.13)	0.11
At 12 months	0.66 (0.18)	0.70 (0.11)	
*p*	0.47	0.10	
FEV1 ≥ 80%, *n* (%)	12 (29%)	35 (35%)	0.08
At baseline	15 (34%)	33 (46%)	
At 12 months	0.61	0.42	
*p*			
ACQ			
At baseline	2.88 (1.28)	2.72 (1.36)	0.31
At 12 months	1.6 (1.13)	1.71 (1.34)	
∆ from baseline	−1.22	−0.95	
*p*	< 0.0001	< 0.0001	
ACQ ≤ 1.5, *n* (%)			
At baseline	7 (18%)	14 (24%)	0.19
At 12 months	27 (60%)	32 (51%)	
*p*	< 0.001	< 0.01	
*Biomarkers*			
Blood eosinophils, cells × 10^9^/L			
At baseline	0.28 (0.16–0.65)	0.25 (0.12–0.51)	< 0.0001
At 12 months	0.24 (0.15–0.47)	0.10 (0.04–0.14)	
*p*	0.41	< 0.0001	
FeNO, ppb			
At baseline	20 (12–35)	24 (11–45)	0.25
At 12 months	14 (9–18)	19 (10–50)	
*p*	0.0009	0.12	
IgE, IU/mL			
At baseline	325 (150–1165)	224 (76–586)	< 0.001
At 12 months	68 (22–698)	197 (85–330)	
*p*	0.001	0.61	

*Note:* Values are mean (SD) or median (p25–p75), unless otherwise stated.

## 4 Discussion

In this real‐life nationwide complete cohort of patients initiating biologics for severe asthma, 17% had comorbid atopic dermatitis. These patients were characterised by an allergic phenotype of asthma, with childhood onset of disease and higher total IgE levels. Prescription patterns of biologics varied between patients with and without comorbid atopic dermatitis, both before and after dupilumab became available. In patients with comorbid atopic dermatitis, elevated IgE levels were determining for first‐line prescription of dupilumab, whereas in patients without comorbid atopic dermatitis, elevated FeNO levels were the biomarker determinant.

In our cohort of patients receiving biologics for severe asthma, atopic dermatitis was a common comorbidity, with 17% of patients being affected. When compared to other severe asthma cohorts, this proportion varies greatly. In the United Kingdom Severe Asthma Register, 5% of patients with severe asthma also have comorbid atopic dermatitis [20] whereas this proportion is 12% for patients registered in the German severe asthma register [21]. In a collective ISAR cohort of patients with severe asthma from 22 countries, 10% are reported to have comorbid atopic dermatitis [22], and in the countries contained in the SHARP central, the proportion of patients with severe asthma and comorbid atopic dermatitis varies between 0% and 14% [23].

A comparison of patterns for first‐line prescription of biologics and subsequent switches between countries and cohorts remains very difficult because the availability of biologics and criteria for initiation and switching vary greatly between countries [24]. However, a previous study examined prescription and switch patterns in patients in ISAR and CHRONICLE and included more than 3000 patients [25]. They found that comorbidity in the form of nasal polyposis was associated with switching biologics and not atopic dermatitis. This is contrary to what we observed in our study; however, the ISAR/CHRONICLE study did not include data from after 2020, and timing can be expected to be a significant factor in prescription patterns of biologics, owing to its impact on the availability of different biologics.

To our knowledge, this is the first study to investigate real‐world prescription patterns of biological therapy in patients with severe asthma and comorbid atopic dermatitis in a complete and nationwide cohort of patients with severe asthma. As there is a free choice between biologics in Denmark and no established rules for switching biologics, DSAR is a valuable platform for investigating prescription patterns in real life. However, there are limitations to our data. In this study, we compared patients in DSAR with and without physician‐assessed comorbid atopic dermatitis; however, the registry does not contain data on the disease severity of atopic dermatitis. In relation to biological therapy, disease severity is very important, as the severity of atopic dermatitis spans from dry skin that requires treatment with emollients to severe, debilitating diseases that require systemic immunosuppressants. The diagnosis of comorbid atopic dermatitis in DSAR is assessed by an asthma specialist and not a dermatologist, and patients are not necessarily evaluated according to the diagnostic criteria. Furthermore, as DSAR is a real‐world register, there will inevitably be missing data, and as treatment is not randomised, data are not appropriate for head‐to‐head comparisons between biologics.

In this study, we showed that patients with comorbid atopic dermatitis receiving biological therapy for severe asthma more often displayed a classic phenotype of allergic asthma with childhood onset, as opposed to patients without comorbid atopic dermatitis who had later onset of disease and fewer had allergic sensitisation, and it is possible that early onset asthma with comorbid atopic dermatitis and allergy represents a distinct endotype within severe asthma. This difference in endotype was also reflected in the prescription pattern of biologics, as more patients with comorbid atopic dermatitis were prescribed omalizumab. In our cohort, 85% of patients with severe asthma and comorbid atopic dermatitis also had allergic rhinitis, a notably high prevalence. In contrast, a meta‐analysis found that allergic rhinitis affects 40% of patients with atopic dermatitis, with only 14% having both asthma and allergic rhinitis [26]. This suggests that our cohort may have an especially high T2 inflammatory drive, consistent with the severity of asthma requiring biological therapy. The combination of allergic rhinitis, atopic dermatitis, and asthma may represent a distinct phenotype of severe T2 inflammation, potentially placing patients at higher risk of severe asthma. However, this remains speculative, as our data do not account for the timing of disease onset. We also observed differences in biomarkers with patients with comorbid atopic dermatitis having higher total IgE compared to patients without atopic dermatitis. This is perhaps unsurprising, as elevated IgE is associated with atopic dermatitis and correlates with disease severity. More surprisingly, we found that patients with comorbid atopic dermatitis had lower FeNO levels than patients without atopic dermatitis. Interestingly, the opposite might be expected, as FeNO is considered a biomarker of IL13 activity [27, 28]. A higher IL13 drive could reasonably be anticipated in patients with comorbid atopic dermatitis, especially given that, unlike asthma, this condition has biologics targeting only IL13 specifically approved for treatment. This could be due to saturation of the IL13 pathway in these patients, or it could indicate differences in epithelial activation according to atopic dermatitis status.

In patients with comorbid atopic dermatitis, elevated IgE levels were the key biomarker determinant for first‐line dupilumab prescription, indicating that it is prescribed in individuals with more severe manifestations of atopic dermatitis. This suggests that higher IgE levels, reflective of greater disease severity of atopic dermatitis, guide treatment decisions in this population. Conversely, in patients without comorbid atopic dermatitis, FeNO was the primary biomarker determining first‐line dupilumab prescription, consistent with its role in predicting therapeutic response to dupilumab in asthma [11].

In this study, the effects of dupilumab on asthma‐related outcomes in the subgroup of patients with comorbid atopic dermatitis were comparable to those of other biologics. This indicates that, in this subgroup, there was no additional benefit of prescribing dupilumab over other biologics for asthma. We also observed that patients with comorbid atopic dermatitis were more likely to switch biologics, with dupilumab chosen most often as the second agent. This could suggest that the decision to switch may have been driven not solely by suboptimal asthma control, but rather by insufficient management of atopic dermatitis, and this remains speculative as atopic dermatitis severity is not captured in DSAR. Still, the most important question remains unanswered: Is there an additional benefit of dupilumab prescription for severe asthma on atopic dermatitis‐related outcomes? It remains an important research goal to investigate collateral effects on other T2‐driven diseases when using biological therapy for severe asthma and vice versa.

## 5 Clinical Implications

Our findings highlight the importance of comorbid atopic dermatitis in shaping treatment decisions for severe asthma. For clinicians, especially those in dermatology and pulmonology, this underscores the need for integrated assessment when selecting biologic therapies. Given the shared inflammatory pathways, multidisciplinary approaches and closer cross‐specialty collaboration may enhance treatment outcomes. These insights also call for structured guidelines and tools to support biologic selection in patients with multiple T2‐driven diseases.

## 6 Conclusion

Dupilumab is more commonly prescribed in patients with severe asthma with comorbid atopic dermatitis than in other patients with severe asthma. In patients with comorbid atopic dermatitis, elevated IgE is determining for first‐line dupilumab prescription, indicating that atopic dermatitis severity is taken into account by prescribing asthma specialists. More research on how biological treatment can be applied to treat coexisting T2 diseases simultaneously is needed.

## Author Contributions

Conceptualisation: all authors. Methodology: all authors. Data curation and formal analysis: M.B. Soendergaard, S. Hansen, K.E.J. Håkansson. Project administration and supervision: M.B. Soendergaard, S. Hansen, K.E.J. Håkansson, C. Porsbjerg. Writing: M.B. Soendergaard (first draft); all authors (final version).

## Conflicts of Interest

M.B.S. has received lecture fees from GSK, AstraZeneca, and ALK. S.H. has received lecture fees from AstraZeneca. K.E.J.H. has received unrestricted research grants from AstraZeneca and Sanofi Genzyme, paid to the institution, and lecture fees from AstraZeneca, Teva, GSK, and Sanofi Genzyme. A.‐S.B. has received lecture fees from AstraZeneca and GSK. A.B. has received lecture fees from AstraZeneca, Novartis, and GSK, and attended advisory boards for AstraZeneca and Novartis. L.M.R. has received lecture fees from AstraZeneca, GSK, and Teva, and support for attending meetings and/or travel from AstraZeneca and Chiesi. S.F.T. has served as a speaker and/or advisor for the following companies: Sanofi, AbbVie, LEO Pharma, Pfizer, Eli Lilly, Novartis, UCB Pharma, Almirall, Union Therapeutics, Symphogen, and Janssen Pharmaceuticals. C.S.U. has received lecture fees from AstraZeneca, GSK, Teva, Sanofi, Orion Pharma, Novartis, Berlin Chemie, Pfizer, and Chiesi. C.P. has received lecture fees from AstraZeneca, GSK, Novartis, Teva, Sanofi, and Chiesi, and participated on a data safety monitoring board or advisory board for AstraZeneca, Novartis, Teva, Sanofi, and ALK. The rest of the authors declare that they have no relevant conflicts of interest.

## Data Availability

The data that support the findings of this study are available on request from the corresponding author. The data are not publicly available due to privacy or ethical restrictions.
